# Intermediate weight changes and follow-up of dietetic treatment in primary health care: an observational study

**DOI:** 10.1186/s40795-020-00377-0

**Published:** 2020-11-16

**Authors:** Lisa D. M. Verberne, Chantal J. Leemrijse, Markus M. J. Nielen, Roland D. Friele

**Affiliations:** 1 Nivel, Netherlands Institute for Health Services Research, P.O. Box 1568, Utrecht, 3500 BN The Netherlands; 2 Tilburg School of Social and Behavioral Sciences, Tilburg University, Tranzo, P.O. Box 90153, Tilburg, 5000 LE The Netherlands

**Keywords:** Overweight, Obesity, Weight loss, Nutritionists, Primary health care

## Abstract

**Background:**

Primary health care data have shown that most patients who were treated for overweight or obesity by a dietitian did not accomplish the recommended treatment period. It is hypothesised that a slow rate of weight loss might discourage patients from continuing dietetic treatment. This study evaluated intermediate weight changes during regular dietetic treatment in Dutch primary health care, and examined whether weight losses at previous consultations were associated with attendance at follow-up consultations.

**Methods:**

This observational study was based on real life practice data of overweight and obese patients during the period 2013–2017, derived from Dutch dietetic practices that participated in the Nivel Primary Care Database. Multilevel regression analyses were conducted to estimate the mean changes in body mass index (BMI) during six consecutive consultations and to calculate odds ratios for the association of weight change at previous consultations with attendance at follow-up consultations.

**Results:**

The total study population consisted of 25,588 overweight or obese patients, with a mean initial BMI of 32.7 kg/m^2^. The BMI decreased between consecutive consultations, with the highest weight losses between the first and second consultation. After six consultations, a mean weight loss of − 1.5 kg/m^2^ was estimated. Patients who lost weight between the two previous consultations were more likely to attend the next consultation than patients who did not lose weight or gained weight.

**Conclusions:**

Body mass index decreased during consecutive consultations, and intermediate weight losses were associated with a higher attendance at follow-up consultations during dietetic treatment in overweight patients. Dietitians should therefore focus on discussing intermediate weight loss expectations with their patients.

## Background

In Europe, almost all primary health care systems provide services for the prevention and treatment of overweight and obesity (i.e. having a body mass index (BMI) ≥ 25 kg/m^2^) [1]. In the Netherlands, general practitioners (GPs), practice nurses, and dietitians are the main healthcare professionals to provide these services. Weight management tasks by GPs and practice nurses may consist of regular weight measurements and advisement on nutrition and physical activity [2, 3]. For more intensive guidance on nutritional health care, patients are advised to consult a dietitian. Dietitians are important health care professionals for providing nutritional health care to overweight and obese patients, with the primary aim to achieve and maintain weight loss [4, 5]. Intensive weight management improves clinical outcomes, and has the potential to reduce complications of diabetes mellitus type 2 [6].

Data from Dutch primary health care show that most patients who visit a dietitian are referred by their GP and approximately half of these patients are diagnosed with overweight or obesity [7]. Further studies on these data demonstrated that overweight patients who were treated by a dietitian lost approximately one BMI point, corresponding to a weight loss of 3.5% of initial body weight [8, 9]. However, most patients did not reach the weight loss goal of ≥5% of initial body weight, and did not accomplish the recommended treatment duration of at least 1 year, as recommended in the guidelines for dietitians.

The effectiveness of weight loss treatment is affected by the combined effects of several factors, including treatment adherence, which is defined as the extent to which a person’s behaviour corresponds with the agreed recommendations from the health care provider [10], sociodemographic factors, and physiological responses during weight loss. Previous studies in primary health care settings found patients’ health status, sex, age, and socio economic status to be important determinants in health care utilisation [11–13]. Similar determinants were indicated in a meta-analysis of adherence to weight loss interventions, which showed that a poor health, a lower age, and a lower socio economic status were associated with a lower adherence [14]. Several other studies have indicated early weight loss as a predictor for lower drop-out rates in weight loss programs [15–21], suggesting that a slow rate of weight loss might discourage patients from continuing with treatment. The current study evaluated real life practice data to examine the degree of weight loss during follow-up of dietetic treatment and its association with attendance at follow-up consultations.

## Methods

### Study design

This observational study was based on routinely recorded data by Dutch dietetic practices that participated in the Nivel Primary Care Database (Nivel-PCD) within the period 2013–2017. The Nivel-PCD contains anonymised patient data from electronic health records, extracted from software programmes used by primary care dietetic practices, as previously described by Verberne et al. [9].

### Study population

All electronic health records were selected from the Nivel-PCD for patients ≥18 years who had a recorded diagnosis of being overweight or obese (BMI ≥ 25 k/m^2^), and who started a treatment with the dietitian between January 2013 and December 2016. Patients were excluded if they had an additional recorded diagnosis for which weight loss might not be the goal for treatment, e.g. for gestational diabetes.

### Measures

Information on sex, age, BMI, dietetic diagnoses, four digit-postal codes of the patient’s neighbourhood, and consultation dates, were derived from the electronic health records of the patients. A variable with three categories was established that defined whether a patient had other recorded dietetic diagnoses 1) no other recorded diagnosis; 2) a recorded diagnosis of diabetes mellitus type 2, hypertension, and/or hypercholesterolemia; and 3) a recorded diagnosis other than diabetes mellitus type 2, hypertension, or hypercholesterolemia. The neighbourhood social status score in 2014 was obtained from the Netherlands Institute for Social Research (SCP) [19]. This is a composite measure on the four digit-postal code level, established with four indicators, i.e. mean income, the proportion of people with a low education level or a low income, and unemployment. For the present study, the social status score was categorised according to the quartiles of the status score in the Netherlands.

### Statistical analyses

Statistical analyses were performed using STATA 14.2. Descriptive statistics were used to present patient characteristics. All recorded one to one consultations were counted that took place within 1 year after start of the treatment to calculate the attrition rate. A multilevel linear regression analysis was performed to estimate the mean changes in BMI during six consecutive consultations, using data from all patients who had a recorded BMI at the first consultation (the initial BMI) and an available BMI measure at one or more of the five following consultations. The initial BMI was used as reference to calculate the change in BMI at each time point. Random intercepts were included to account for clustered data of patients within dietetic practices and for repeated measurements within patients.

Weight changes between two consecutive consultations were calculated by subtracting the BMI recorded at the first consultation from the BMI recorded at the last consultation for all patients with available BMI measurements. Subsequently, three categories of weight change were established. The category “no weight loss” (change in BMI: ≥ 0 kg/m^2^) was used as a reference. Furthermore, two categories of weight loss were created: “moderate weight loss”, and “high weight loss”. The cut off value for these two categories was based on the median weight loss between consultations, which was approximately − 0.5 kg/m^2^. We used a multilevel logistic regression, including a random intercept to account for clustered data of patients (level 1) within dietetic practices (level 2), to calculate the odds ratios for attendance at consultation 3, 4, 5, and 6, across the categories of weight change between consultation 1 & 2, consultation 2 & 3, consultation 3 & 4, and consultation 4 & 5, respectively. A second model was used to control for potential confounding factors, and included variables for sex, age, initial BMI, dietetic diagnosis, and social status score. A two-tailed *P*-value of < 0.05 was considered statistically significant.

## Results

**Fig. 1:**
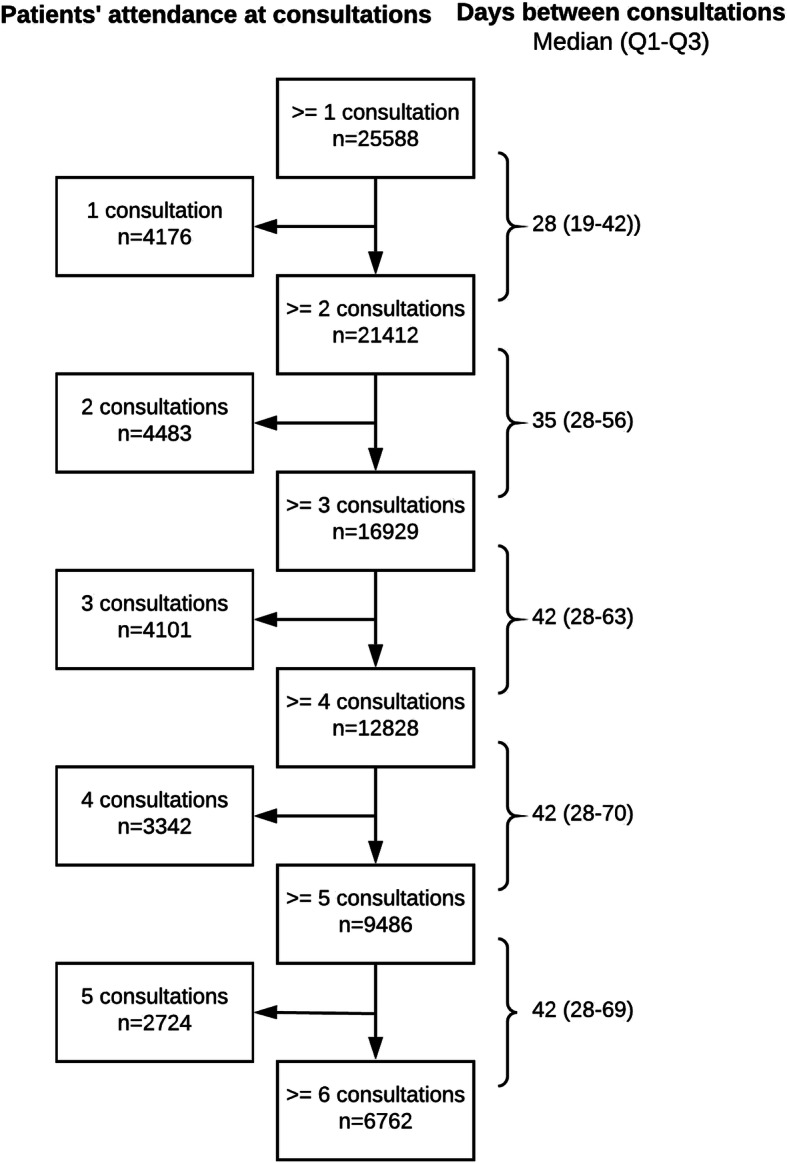
Attrition rate during dietetic treatment

**Fig. 2:**
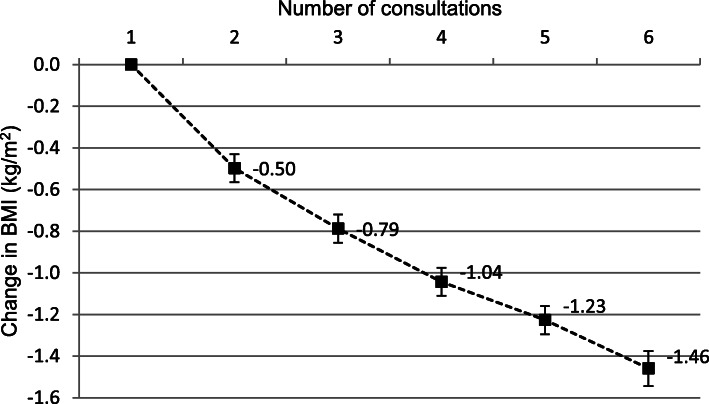
Mean change in body mass index (BMI) between six consecutive consultations. Means are adjusted for clustered data of patients within dietetic practices, and for repeated measurements within patients. The error bars represent the 95% confidence intervals around the means

The total study population consisted of 25,588 patients from 77 dietetic practices. Table 1 shows the characteristics of the study population. Patients were on average 54 years old, had a mean BMI of 32.7 kg/m^2^ at the start of treatment, and 64% were female. Sixteen percent of the 25,588 patients dropped out after one consultation and approximately a quarter of the patients attended six consultations or more, corresponding to a mean treatment duration of 6.5 months (Fig. 1). The time between consultations increased from 28 days between the first and second consultation to 42 days after the third consultation. For 79% of the study patients, a BMI was recorded at the first consultation (i.e. the initial BMI). At each following consultation, BMI was recorded for approximately 70% of the patients. The BMI decreased during consecutive consultations, with the highest weight losses occurring between the first two consultations. After six consultations, a mean weight loss of − 1.5 kg/m^2^ was estimated (Fig. 2). This is equivalent to a weight reduction of 4% of initial weight (average initial weight 95.9 kg). A similar weight loss pattern was shown in additional analyses that included only patients who attended six or more consultations with a recorded BMI at each consultation. Table 2 shows the association of attendance at a consultation with the weight change between the two previous consultations. Patients who lost weight between the two previous consultations were more likely to attend the next consultation than patients who did not lose weight or gained weight. These associations were present in both the crude and adjusted models.

**Table 1:** Patient characteristics (*N* = 25,588)

	Percent/Mean (SD)
Sex (female)	63.9
Age (years)	53.7 (15.2)
Initial body mass index (kg/m^2^)	32.7 (5.4)
Initial body weight (kg)	95.9 (18.5)
Dietetic diagnosis	
No other diagnosis	38.9
Diagnosis of diabetes mellitus type 2, hypertension, and/or hypercholesterolemia	48.3
A diagnosis other than diabetes mellitus type 2, hypertension, or hypercholesterolemia	12.9
Neighbourhood social status score:	
Quartile 1—low	36.7
Quartile 2	23.9
Quartile 3	18.1
Quartile 4—high	21.3

**Table 2:** The association of attendance at a consultation with weight change between the two previous consultations

	Category of weight change between two previous consultations		
	no weight loss	moderate weight loss	high weight loss
Attendance at 3rd consultation (yes/no)^a^	2548/705	3322/706	4810/864
Model 1	ref.	1.28 (1.14–1.44)	1.48 (1.33–1.67)
Model 2	ref.	1.28 (1.14–1.44)	1.40 (1.25–1.57)
Attendance at 4th consultation (yes/no)^b^	2363/762	2914/662	2803/639
Model 1	ref.	1.40 (1.24–1.58)	1.40 (1.24–1.58)
Model 2	ref.	1.44 (1.27–1.64)	1.35 (1.19–1.54)
Attendance at 5th consultation (yes/no)^c^	2131/786	2100/527	1844/497
Model 1	ref.	1.43 (1.26–1.62)	1.33 (1.17–1.52)
Model 2	ref.	1.40 (1.21–1.60)	1.27 (1.11–1.47)
Attendance at 6th consultation (yes/no)^d^	1739/735	1511/422	1035/357
Model 1	ref.	1.44 (1.25–1.66)	1.21 (1.04–1.41)
Model 2	ref.	1.46 (1.25–1.70)	1.17 (0.99–1.38)

Odds ratios are presented with their 95% confidence intervals

Model 1: adjusted for clustered data of patients within dietetic practices

Model 2: model 1+ adjustment for sex, age, initial body mass index (BMI), dietetic diagnosis, and social status score

^a^ Weight loss category according to change in BMI between consultations 1 & 2

^b^ Weight loss category according to change in BMI between consultations 2 & 3

^c^ Weight loss category according to change in BMI between consultations 3 & 4

^d^ Weight loss category according to change in BMI between consultations 4 & 5

## Discussion

### Main findings

This study evaluated intermediate weight changes during regular dietetic treatment and its association with attendance at follow-up consultations in Dutch primary health care. The study elaborates on our previous study which showed that a higher weight loss was associated with a longer treatment time [9]. The present study showed that 16% of the patients only had one consultation, which is corresponding to findings of an Italian study that reported a dropout rate of 21% after 1 month in an obesity treatment programme in a clinical setting [16]. Furthermore, we showed that the mean change in BMI between consultations decreased from approximately − 0.5 kg/m^2^ at the second consultation to approximately − 0.25 kg/m^2^ at the following consultations, which is in accordance with reviews on clinical trials that also observed a diminishing trend for weight loss over time [20–22]. We found intermediate weight losses during dietetic treatment in primary health care to be associated with a higher attendance rate at the next consultation. Similar findings have also been shown in other studies in real life settings, other than primary health care [16, 18], and in studies on dietary weight loss interventions, demonstrating an association of early weight loss with the dropout rate [15, 17, 23–25]. For example, Batterham et al. found that people with a weight loss ≤2% were five times more likely to dropout from a weight loss trial than those with a weight loss > 2% in the first month [15].

### Strengths and limitations

We used a large database with real life practice data from overweight and obese patients treated by dietitians in Dutch primary health care. This database includes all information relevant for reimbursement and treatment purposes. In addition, we could link data on social status score to the patient records. We were therefore able to control for important variables related to health care utilisation.

A limitation of the study was the availability of anthropometric data. Measurements of BMI were not recorded at all consultations or for all patients. Additional analyses showed that patients who attended the next consultation were more likely to have had their BMI recorded during the previous consultation (approximately 70%) than patients who did not attend the next consultation (approximately 60%) (data not shown). We could, however, not check whether missing data on BMI were affected by disappointing weight loss results, or by software issues, or recording habits of the dietitian. For further research it would be interesting to enhance the dataset with information on diagnostic measurements and drug prescriptions from electronic health data of general practices.

Another issue that needs consideration is that we only evaluated attendance at follow-up consultations, which does not necessarily mean that a patient is following the instructions for lifestyle changes as recommended by their dietitian. Information about the patients’ compliance with the recommended lifestyle changes by their dietitian was not available in our database. Furthermore, we were not able to evaluate the dietitian-patient relationship, which has been shown to have an important role in adherence to nutritional treatment [26].

### Implications of the findings

We showed that the BMI of overweight and obese patients who were treated by dietitians decreased between consecutive consultations, with the highest weight loss between the first and second consultation. This early weight loss is important, since it has been associated with successful weight loss and weight maintenance in the long-term [17, 27–29]. However, after these first consultations, dietitians should focus on discussing realistic weight loss expectations with their patients. Refining intermediate weight loss goals would possibly help to improve the continuation of treatment. Furthermore, a higher frequency of consultations with a dietitian might aid an earlier intervention in patients who are not complying with the advice from their dietitian [21, 30]. As supported by Stubbs et al. [31], future research is recommended to examine treatments that are sensitive to patients’ individual needs. Possibly an algorithm can help to identify and assess patients for their optimal therapy [32].

Participation in a weight loss program requires a long-term investment from patients. In the present research we showed that BMI decreased during consecutive consultations with a dietitian. The majority of the patients, however, did not attend more than six consultations, and did not reach clinically relevant weight loss goals. In contrast to most previous studies that have demonstrated the effectiveness of weight loss programs in interventional trials, with probably highly motivated patients, we studied dietetic treatment in real life practice where financial and environmental factors play an important role. To illustrate, in the Netherlands, generally, only 3 h of dietetic healthcare are covered by the basic health insurance, and it has been shown that limiting the reimbursement of dietetic treatment resulted in fewer patients visiting the dietitian, since many patients cannot or are unwilling to pay for dietetic treatment [33]. We, therefore, agree with Wadden et al. [34], that more research is needed to find effective methods for weight loss treatment in primary care, also taking into account financial and environmental factors.

## Conclusions

This study was conducted to gain a greater understanding of the adherence to dietetic treatment in primary health care. We demonstrated that the BMI of overweight and obese patients who were treated by dietitians in primary health care decreased between consecutive consultations, and found that intermediate weight losses during dietetic treatment were associated with a higher attendance at follow-up consultations. In order to improve the retention rate of patients during dietetic treatment, dietitians should focus on discussing intermediate weight loss expectations with their patients.

## Data Availability

The datasets used in the current study are available from the corresponding author on reasonable request.

## Abbreviations

BMI: Body mass index
Nivel-PCD: Nivel Primary Care Database
